# Draft genome assembly of *Colletotrichum musae*, the pathogen of banana fruit

**DOI:** 10.1016/j.dib.2018.01.002

**Published:** 2018-01-08

**Authors:** Wilson José da Silva Junior, Raul Maia Falcão, Lucas Christian de Sousa-Paula, Nicolau Sbaraini, Willie Anderson dos Santos Vieira, Waléria Guerreiro Lima, Sérgio de Sá Leitão Paiva Junior, Charley Christian Staats, Augusto Schrank, Ana Maria Benko-Iseppon, Valdir de Queiroz Balbino, Marcos Paz Saraiva Câmara

**Affiliations:** a Departamento de Agronomia, Universidade Federal Rural de Pernambuco, Recife, PE, Brazil; b Departamento de Genética, Universidade Federal de Pernambuco, Recife, PE, Brazil; c Programa de Pós-Graduação em Biologia Celular e Molecular, Universidade Federal do Rio Grande do Sul, RS, Brazil; d Faculdade Guararapes, Pernambuco, PE, Brazil

## Abstract

*Colletotrichum musae* is an important cosmopolitan pathogenic fungus that causes anthracnose in banana fruit. The entire genome of *C. musae* isolate GM20 (CMM 4420), originally isolated from infected banana fruit from Alagoas State, Brazil, was sequenced and annotated. The pathogen genomic DNA was sequenced on HiSeq Illumina platform. The *C. musae* GM20 genome has 50,635,197 bp with G + C content of 53.74% and in its present assembly has 2763 scaffolds, harboring 13,451 putative genes with an average length of 1626 bp. Gene prediction and annotation was performed by Funannotate pipeline, using a pattern for gene identification based on BUSCO.

**Specifications Table**

**Table:** 

Subject area	*Biology*
More specific subject area	*Microbiology, Agricultural, Genomics.*
Type of data	*Genome sequence data*
How data was acquired	*Illumina HiSeq. 2500 Next Generation Platforms*
Data format	*Assembled genome sequence.*
Experimental factors	*Genomic DNA was extract from mycelial growth in culture medium.*
Experimental features	*Genome of Colletotrichum musae strain GM20 was sequenced and assembled.*
Data source location	*Colletotrichum musae strain GM20 was isolated from banana lesions, in Maceio, Pernambuco Brazil.*
Data accessibility	*The Colleotrichum musae GM20 genome is available in DDBJ/ENA/GenBank under the accession number NWMS01000000.*
Related research article	
Data accessibility	https://www.ncbi.nlm.nih.gov/nuccore/NWMS00000000

**Value of the Data**

• *Colletotrichum musae* is the causal agent of anthracnose in banana fruits, the main disease post-harvest worldwide.
• This is the first genome sequence of *Colletotrichum musae* using next-generation sequencing available in public database.
• The published genome data herein will facilitate biology, pathogenicity, evolution and interaction pathogen-host studies of *Colletotrichum musae*, through comparative genomes studies of *Colletotrichum* spp. and related species.

## 1 Data

Fungi infection in plants is the most frequent cause of extensive loses in Agriculture. The fact that many endophytic fungi can case infection adds further complexity to fungal plant pathogens. Banana (*Musa* sp.) is one of the world's important food crops and a staple food for more than 400 million people [1]. Over 100 million tons are produced worldwide at some 5 million hectares and the cultivated area is expected to increase in the future [2]. However, banana fruits are highly susceptible to pathogens, and anthracnose disease caused by fungi from *Colletotrichum* genus is amongst the most frequents. *Colletotrichum* comprises over 100 species that are able to infect and damage diverse crops around the world [3].

Due to its ubiquity, substantial destruction capacity and scientific importance as a model of pathosystems, *Colletotrichum* spp. are among the top 10 of most important plant pathogens according to the international community of plant pathology researchers [4]. *Colletotrichum musae* (Berk. and M.A. Curtis), the causative agent of anthracnose, is a major post-harvest pathogen of banana fruits and causes severe global crop losses [5]. The disease develops from a latent fungal infection during pre-harvest, originated from spores that are present in immature fruits in the field. Symptoms, such as patches on the bark (brown to black color) and depressed lesions, appear in the ripening of the fruits. Furthermore, under high humidity, the formation of salmon-colored acervuli can be observed [6]. The infection thus accounts for a reduction in fruit viability during maturation, transport and storage periods [7], leading to a commercial depreciation and shortening fruit's shelf life.

To circumvent post-harvest losses, chemical fungicides are usually adopted, but other side-methods (e.g., radiation treatment, hot water removal, refrigeration, induced resistance and biological control agents) have also been applied [8]. However, chemical fungicide usage has been limited by potential harmful effects to human health and environment. Besides, fungal pathogens are known to quickly develop resistance to chemical defensives [9].

Furthermore, the absence of available genomic sequences from *C. musae* is one of the main limitations for best characterization of fungal virulence determinants and development of improved management strategies. Here we report, for the first time, the whole genome sequence of the *C. musae* strain GM20 (CMM 4420) isolated from infected banana fruit from Alagoas, Brazilian Northeast State.

In recent years, several phytopathogenic fungal genomes have been published boosting the discovery of virulence determinants in these species. Expectedly, our analysis will encourage further studies of *C. musae* biology, which should provide better details about host-pathogen interaction, leading to new management measures.

## 2 Experimental design, materials, and methods

### 2.1 DNA extraction and genome sequence

The GM20 isolate of *C. musae* was cultured, and DNA was extracted as previously described [10]. Whole shotgun genome sequence of *C. musae* GM20 was generated using the Illumina HiSeq. 2500 platform (Illumina, San Diego, CA) at the Center for Functional Genomics - Universidade de São Paulo (Piracibaba, Brazil). The libraries were prepared with the Illumina Nextera XT DNA Library Prep Kit (Illumina, San Diego, CA) and the sequencing was performed on a HiSeq Flow Cell v4 with HiSeq SBS Kit v4 (Illumina, San Diego, CA), leading to 100 bp paired-reads (2×).

### 2.2 De novo assembly and genes annotation

The shotgun sequencing produced 13,273,851 paired reads. Initially, FastQC [11] was applied to analyse reads quality, and adapters were trimmed using FASTX-Toolkit 0.0.13 (http://hannonlab.cshl.edu/fastx_toolkit). Originally, three assemblers were tested: ABySS 2.0.2 [12]; SPAdes 1.10 [13]; Velvet 1.1 [14], with SPAdes showing the best results (12,435 contigs >500 bp). Additionally, Redundans [15] posteriorly ran for scaffolds assembly.

Assembly statistics were generated by QUAST 3.9 (Table 1) [16]. Gene prediction and annotation was carried out with Funannotate pipeline [17] BUSCO 2.0 [18] [parameters: Sordariomycetes database (*Verticillium longporum* selected as closely-related species)] to generate the training files for two genome predictors: GeneMark-ES [19] and AUGUSTUS [20]. Moreover, BUSCO 2.0 was employed to evaluate genome completeness, based on conservation of single-copy benchmarking universal single-copy orthologs (BUSCOs).

**Table 1:** Genome assembly statics for *Colletotrichum musae* GM20.

	*C. musae* GM20
Assembly size	50.7 Mb
Coverage sequencing	100×
Sequencing technology	Illumina HiSeq. 2500
Number of scaffolds	2763
N50 scaffolds length	32,818
Number of contigs	10,618
Number of predicts genes	13,451
Overall GC content	53.74
Public access to genome	NWMS01000000

The final assembly of the *C. musae* GM20 genome was determined to be 50,635,197 bp with a G+C content of 53.74% in 2763 scaffolds (maximum 208,119 bp; N50 32,818 bp), and 13,451 genes were predicted. This whole Genome Shotgun project has been deposited at DDBJ/ENA/GenBank under the accession number NWMS00000000. The version described is this paper is version NWMS01000000.

BUSCO analysis showed a high degree of completeness with a BUSCO score of 96.3%, of which 1263 genes were complete BUSCOs, four were complete duplicated BUSCOs, 23 were fragmented BUSCOs, and 25 were missing BUSCO orthologs out of the 1315 BUSCO groups searched.

## References

[1] Holscher D., Dhakshinamoorthy S., Alexandrov T., Becker M., Bretschneider T., Buerkert A., Crecelius A.C., De Waele D., Elsen A., Heckel D.G., Heklau H., Hertweck C., Kai M., Knop K., Krafft C., Maddula R.K., Matthaus C., Popp J., Schneider B., Schubert U.S., Sikora R. a, Svato A., Swennen R.L. (2014). Phenalenone-type phytoalexins mediate resistance of banana plants (Musa spp.) to the burrowing nematode Radopholus similis. Proc. Natl. Acad. Sci..

[2] FAO, Food and agricultural organization. 〈http://www.fao.org/home/en/〉, 2017 (Accessed 01 Jan 2017).

[3] Cannon P.F., Damm U., Johnston P.R., Weir B.S. (2012). Colletotrichum - current status and future directions. Stud. Mycol..

[4] Dean R., Van Kan J.A.L., Pretorius Z.A., Hammond-Kosack K.E., Di Pietro A., Spanu P.D., Rudd J.J., Dickman M., Kahmann R., Ellis J., Foster G.D. (2012). The Top 10 fungal pathogens in molecular plant pathology. Mol. Plant Pathol..

[5] Maqbool M., Ali A., Ramachandran S., Smith D.R., Alderson P.G. (2010). Control of postharvest anthracnose of banana using a new edible composite coating. Crop Prot..

[6] Ranasinghe L.S., Jayawardena B., Abeywickrama K. (2003). Use of waste generated from cinnamon bark oil (Cinnamomum zeylanicum Blume) extraction as a post harvest treatment for Embul banana. J. Food Agric. Environ..

[7] Slabaugh W.R., Grove M.D. (1982). Postharvest diseases of bananas and their control. Plant Dis..

[8] Zhimo V.Y., Dilip D., Sten J., Ravat V.K., Bhutia D.D., Panja B., Saha J. (2017). Antagonistic Yeasts for Biocontrol of the banana postharvest anthracnose pathogen Colletotrichum musae. J. Phytopathol..

[9] Sonah H., Deshmukh R.K., Bélanger R.R. (2016). Computational prediction of effector proteins in fungi: opportunities and challenges. Front. Plant Sci..

[10] Doyle J.J.D.J.L.J.J., Doyle J.J.D.J.L.J.J. (1990). Isolation of plant DNA from fresh tissue. Focus (Madison).

[11] Andrews S. (2010). FastQC: a quality control tool for high throughput sequence data. Babraham Bioinform..

[12] Simpson J.T., Wong K., Jackman S.D., Schein J.E., Jones S.J.M., Birol I. (2009). ABySS: a parallel assembler for short read sequence data. Genome Res..

[13] Bankevich A., Nurk S., Antipov D., Gurevich A.A., Dvorkin M., Kulikov A.S., Lesin V.M., Nikolenko S.I., Pham S., Prjibelski A.D., Pyshkin A.V., Sirotkin A.V., Vyahhi N., Tesler G., Alekseyev M.A., Pevzner P.A. (2012). SPAdes: a new genome assembly algorithm and its applications to single-cell sequencing. J. Comput. Biol..

[14] Zerbino D.R. (2010). Using the Velvet de novo assembler for short-read sequencing technologies. Curr. Protoc. Bioinform..

[15] Pryszcz L.P., Gabaldón T. (2016). Redundans: an assembly pipeline for highly heterozygous genomes. Nucleic Acids Res..

[16] Gurevich A., Saveliev V., Vyahhi N., Tesler G. (2013). QUAST: quality assessment tool for genome assemblies. Bioinformatics..

[17] J.M. Palmer, Funannotate: a Fungal Genome Annotation and Comparative Genomics Pipeline〈https://github.com/nextgenusfs/funannotate〉, 2016.

[18] Simão F.A., Waterhouse R.M., Ioannidis P., Kriventseva E.V., Zdobnov E.M. (2015). BUSCO: assessing genome assembly and annotation completeness with single-copy orthologs. Bioinformatics.

[19] Besemer J. (2001). GeneMarkS: a self-training method for prediction of gene starts in microbial genomes. Implications for finding sequence motifs in regulatory regions. Nucleic Acids Res..

[20] Stanke M., Steinkamp R., Waack S., Morgenstern B. (2004). AUGUSTUS: a web server for gene finding in eukaryotes. Nucleic Acids Res..

