# Supplementary material for: Draft genome assembly of Colletotrichum musae, the pathogen of banana fruit
Source: Data Brief. 2018 Jan 8;17:256–60. doi: 10.1016/j.dib.2018.01.002 (PMC5790810; doi:10.1016/j.dib.2018.01.002)
Supplement: Supplementary file 1 — Supplementary material [file mmc1.docx]

**Conflict of interest** The authors declare that there is no conflict of interests with respect

to the work published in this paper.
